# Employees’ Perceptions of Social Norms as a Result of Implementing the Participatory Approach at Supervisor Level: Results of a Randomized Controlled Trial

**DOI:** 10.1007/s10926-016-9659-9

**Published:** 2016-08-24

**Authors:** S. M. Ketelaar, F. G. Schaafsma, M. F. Geldof, C. R. L. Boot, R. A. Kraaijeveld, W. S. Shaw, U. Bültmann, J. Twisk, J. R. Anema

**Affiliations:** 1 Department of Public and Occupational Health, EMGO+ Institute for Health and Care Research, VU University Medical Center, Amsterdam, The Netherlands; 2 Research Center for Insurance Medicine, Collaboration Between AMC-UMCG-UWV-VU University Medical Center, Amsterdam, The Netherlands; 3 Institute for Work and Health, Toronto, Canada; 4 Liberty Mutual Research Institute for Safety, Hopkinton, MA USA; 5 University of Massachusetts Medical School, Worcester, MA USA; 6 Department of Health Sciences, Community and Occupational Medicine, University of Groningen, University Medical Center Groningen, Groningen, The Netherlands; 7 Department of Epidemiology and Biostatistics, EMGO Institute for Health and Care Research, VU University Amsterdam, Amsterdam, The Netherlands

**Keywords:** Participatory approach, Workplace, Sick leave, Occupational health, Supervisors

## Abstract

*Purpose* A multifaceted implementation strategy was targeted at supervisors to encourage them to apply a participatory approach (PA) in dealing with employees’ work functioning problems due to health concerns. This paper assesses the effect on employees’ perceived social norms regarding the use of the PA to deal with work functioning problems. *Methods* Three organizations participated in a cluster randomized controlled trial, with randomization at the department level. Supervisors in the PA intervention departments received the implementation strategy consisting of a working group meeting, supervisor training, and optional coaching. Supervisors in the control departments received written information about the PA only. In two of the organizations, employees were invited to complete surveys at baseline and at 6-month follow-up. The primary outcome was perceived social norms regarding the use of the PA to deal with work functioning problems. Secondary measures included attitudes and self-efficacy, and intention regarding joint problem solving, and sick leave data. Effects were analyzed using multilevel analyses to account for nesting of cases. *Results* At baseline, 273 employees participated in the survey, with follow-up analyses of 174 employees. There were no statistically significant group effects on employee outcome measures. The intervention group showed a larger reduction in mean sick days (from 4.6 to 2.4 days) versus the control group (from 3.8 to 3.6 days), but this difference did not reach statistical significance (*p* > .05). *Conclusion* The multifaceted strategy to implement the participatory approach for supervisors did not show effects on outcomes at the employee level. To gain significant effects at the employee level, may require that an implementation strategy not only targets management and supervisors, but also employees themselves.

Trial registration: NTR3733.

## Introduction

For employees dealing with health complaints, this can impede work functioning and result in more sick leave days [1]. One possible method to reduce long-term disability for these employees is to improve organizational communication and problem-solving and detect these health complaints at an earlier stage. However, employees may be reluctant to report health complaints in the workplace for fear of stigma, unfair treatment, or job loss despite on-going struggles [2, 3]. This is especially true of mental health complaints, where workplace disclosure may be more difficult. In these cases, a perceptive and caring supervisor is important to help the employee understand and overcome any work functioning problems due to these complaints through changes in pacing, work methods, and assistance from others, or other forms of job modification [4]. There is a need for organizational interventions to reduce the uncertainty of employees and supervisors about how to best address work functioning problems.

One method for addressing this problem is to support supervisor training in communication and problem-solving. Such a participatory approach (PA) is effective to improve return-to-work (RTW), to shorten the duration of sick leave [5–9] and to reduce various health complaints of employees [10–12]. The PA encompasses a protocol for workplace interventions, in which supervisors and employees separately identify work functioning problems due to health complaints and subsequently discuss and solve these problems together. In this study, two innovative elements are introduced regarding application of the PA. In previous studies, the PA intervention was applied to address barriers for RTW. In the present study, we encourage supervisors to identify and respond to work functioning problems early, thereby aiming to prevent employees from long-term sick leave. A second innovative element of the present study regards the person applying the PA. In previous studies, the PA was applied by an occupational health professional (OHP) as RTW coordinator, acting as process leader. However, supervisors and colleagues are the first to notice that an employee has work functioning problems or is at risk of sick leave. Also, the supervisor is a key player in managing and optimizing work functioning of an employee with health complaints, and in providing the necessary conditions to help the employee to remain at work [13–15]. When applying the PA as a preventive strategy, it seems appropriate that the supervisor is the one to apply the PA instead of an OHP, thus acting as both a process leader and a participant in joint-problem solving together with the employee.

Several barriers at the level of the organization, supervisors and employees may impede implementation of the PA within an organization [16–18]. At the organizational level, the PA might not comply with organizational sick-leave policies and practices. For example, the organization may not encourage employees to discuss work functioning problems with their supervisors. Furthermore, the HR department may not support actual work adaptations to tackle work functioning problems. At the level of supervisors, barriers may be lack of self-efficacy to discuss work functioning problems with employees with health complaints and to jointly solve these problems, lack of the required attitude, and lack of sufficient knowledge about health complaints, the possibilities of work adaptations for employees with health complaints, and when to consult an OHP. As for barriers at the employee level, it is expected that employees may experience a lack of empathy, respect and support from their supervisor. In addition, employees may experience that their supervisor does not provide sufficient possibilities for joint problem-solving regarding work functioning problems [19]. To tackle these barriers, a multifaceted implementation strategy was set up consisting of (1) a working group meeting in each participating organization with relevant stakeholders, (2) a half-day training for supervisors and (3) the possibility for supervisors to receive individual coaching in application of the PA [20].

As a theoretical framework for the randomized controlled trial, the Attitude-Social Influence-Self-efficacy (ASE) model was used [21]. The ASE model assumes that behavior, in this case the supervisor and employee discussing work functioning problems and risk of sick leave, can be predicted by the intention to perform that behavior. This intention is in turn determined by an individual’s attitude, perceived social norms from others, and self-efficacy to perform that behavior [21]. The implementation strategy studied in our trial targeted the organization and supervisors. However, we were also interested in exploring to what extent effects of the implementation strategy might work through to the employee level. As described above, supervisors were positioned as key player and were trained in applying the PA. It was hypothesized that this would encourage supervisors to promote discussions on work functioning problems with their employees. We therefore reasoned that an effect could be expected on employees’ perceived social norms from their work environment regarding the use of the PA, i.e. joint problem-solving of work functioning problems. Other outcomes that we examined on employee level were attitude, self-efficacy and intention regarding joint problem-solving to improve work functioning, sick leave data, perceived supervisor support, satisfaction with regard to discussing reduced work functioning due to health complaints with their supervisor, and whether employees actually did discuss (risk of) sick leave with their supervisor.

## Methods

### Study Design and Participants

Three organizations took part in a cluster-randomized controlled trial (RCT) performed in 2012 and 2013: a steel factory, a university medical center, and a university [20]. In the cluster-RCT, random allocation to either the intervention group or the control group was performed at department level to limit contamination between supervisors and their employees in both groups. Departments within organizations were matched as pairs, based on the number of participating supervisors within the departments and departments’ sick-leave frequencies. Randomization was performed by an independent researcher who was not involved in the study. Researchers, employees, supervisors, managers, human resource professionals (HRPs), and occupational health professionals (OHPs) were not blinded to the intervention. The study protocol was approved by the Medical Ethics Committee of the VU University Medical Center, Amsterdam, The Netherlands. The report of the cluster-RCT followed the Consolidated Standards of Reporting Trials guidelines [20]. The present paper concerns secondary data analyses of outcomes on employee level. Data at employee level were only available for the university medical center and the university, therefore, data from the steel factory could not be included in the analyses.

Employees were eligible for participation if they had a minimum age of 18 years. Employees who had a different supervisor at 6 months’ follow-up compared to baseline were excluded from the analyses.

### Intervention

#### Multifaceted Implementation Strategy

The multifaceted strategy to implement the PA was applied in the intervention group and consisted of three components, following the baseline measurement (month 1): (A) one working group meeting per organization with stakeholder representatives (month 2); (B) supervisor training in application of the PA (months 3); and (C) optional supervisor coaching (month 4–12). The implementation strategy is described in more detail in our design study [20].

The supervisor training (part B of the intervention) included how to identify an employee with work functioning problems or at risk of sick leave, how to discuss the risk of sick leave with the employee, the steps within the protocol on PA application, and how to apply the protocol in daily practice. During this training, supervisors were also encouraged to let their employees know that they had followed this training, and to tell that their door was open for employees who experienced work functioning problems.

The protocol on PA application consisted of seven steps to identify and solve employees’ work functioning problems due to health complaints (Box 1). Although the PA protocol was primarily targeted towards employees at risk of sick leave, supervisors were also instructed to apply the protocol to sick-listed employees, i.e. to jointly identify and solve barriers to RTW. The PA intervention calls for the supervisor to act as both participants (i.e. the supervisory role) and as leader of the PA process. However, if needed, the supervisor or the employee could ask an OHP to act as process leader.

**Box 1:** Protocol for application of PA

Meeting 1	*Step 1*	Supervisor addresses the employee’s work functioning problems due to health complaints or risk of sick leave and informs the employee about the PA protocol
Preparation	*Step 2*	Employee makes an inventory of his or her work tasks and activities, prioritizes work functioning problems regarding these activities, and thinks of possible solutions for the two most important work functioning problems
	*Step 3*	Supervisor makes an inventory of the employee’s work tasks and activities, prioritizes work functioning problems regarding these activities, and thinks of possible solutions for the two most important work functioning problems
Meeting 2	*Step 4*	Supervisor and employee discuss work functioning problems and possible solutions, and assess the applicability of these solutions
	*Step 5*	Supervisor and employee agree on an action plan to realize solutions
Realization	*Step 6*	Solutions are prepared and realized
Meeting 3	*Step 7*	Supervisor and employee evaluate the action plan and the realized solutions

#### Minimal Implementation Strategy

The minimal implementation strategy used in the control group consisted of the distribution of written information about the PA intervention. After completion of the study, supervisors in the control group departments were invited to receive training in the same multifaceted implementation PA strategy.

### Outcomes

All outcome measures were obtained from study participants (employees) at baseline and after 6 months.

We were primarily interested in the effects of the PA intervention on the perceptions of social norms within the organization, especially regarding problem-solving together with supervisors to improve work functioning. Two items were used to measure perceptions of social norms regarding joint problem-solving with the supervisor: “My organization encourages me to address these situations with my supervisor” and “My supervisor expects me to engage in joint problem-solving to improve my work functioning.” Response categories for both items ranged from 1 (totally disagree) to 5 (totally agree).

Effects on several other employee outcome measures were also explored. First of all, employees’ attitudes, self-efficacy and intention regarding joint problem-solving to improve work functioning were assessed. Response categories for all items ranged from 1 (totally disagree) to 5 (totally agree) and for each outcome with >1 item, the sum score was calculated. Attitude was measured using three items (total score range 3–15) (Cronbach’s alpha = .74), for example “In these situations it is important to inform your supervisor in time”. Self-efficacy (total score range 3–15) was measured with three items (Cronbach’s alpha = .86), for example “I have mastered the skills to address these situations with my supervisor”. Intention was measured with one item (score range 1–5): “It is very likely that I will engage in joint problem-solving together with my supervisor to improve my work functioning”.

In addition, self-efficacy regarding return-to-work was measured using four selected items from the 19-item Return-to-Work Self-Efficacy Questionnaire (RTWSE-19) [22] relevant to the purpose of our intervention (Cronbach’s alpha = .88), for instance “How confident are you regarding your ability to suggest work adaptations to your supervisor, to reduce your health complaints?” Response categories for all items ranged from 1 (not at all confident) to 10 (totally confident), with a total score range of 4–40.

Furthermore, employees were asked to report how often they had called in sick during the last 6 months and how many work days they were sick-listed in total in the last 6 months.

Perceived supervisor support and satisfaction were also assessed. Perceived supervisor support was measured using the Dutch version of the Supervisor Social Support scale of the Job Content Questionnaire [23]. This scale consists of four items (Cronbach’s alpha = .84), for instance “My supervisor pays attention to the wellbeing of his/her employees.” Response categories ranged from 1 (totally disagree) to 4 (totally agree), with a total score range of 4–16. Satisfaction with regard to discussing impaired work functioning or (risk of) sick leave due to health complaints with their supervisor was measured with one item “How satisfied are you regarding discussing your reduced work functioning or (risk of) sick leave due to health complaints with your supervisor?”. Response categories ranged from 1 (very dissatisfied) to 5 (very satisfied).

Lastly, participants were asked whether they had discussed work functioning problems or (risk of) sick leave with their supervisor. In addition, they were asked whether a third party had been present at a meeting between the participant and their supervisor.

#### Possible Confounders and Effect Modifiers

Several factors at the employee level were taken into account as possible confounders or effect modifiers: age, sex, level of education, job insecurity [23] (3 items), organization (university or university medical center), general health [24] (1 item; 1 = excellent, 5 = poor), being at risk for sick leave at baseline (2 items; yes/no), being at sick leave at baseline (yes/no), distress [25] (16 items; 0 = no, 4 = very often), need for recovery [26] (11 items; yes/no), decision authority [23] (3 items; 1 = completely disagree, 4 = completely agree), perceptions of supervisors’ leadership style [27] (transformational leadership; 7 items; 1 = (almost) never, 5 = (almost) always).

### Statistical Analyses

Intention-to-treat analyses were performed at the employee level. Baseline characteristics were presented using descriptive statistics. A drop-out analysis was performed to determine whether non-completers and completers (i.e. those who filled out both questionnaires and those who did not) differed on perceived social norms at baseline, using a Mann–Whitney *U* test. Multilevel analyses were performed for all outcome variables with the employee clustered within the supervisor, who is in turn clustered within the department. All analyses were adjusted for the baseline value of the particular outcome. Both crude analyses and analyses adjusted for abovementioned confounders were performed. Effects on perceived social norms were further examined with two sub-group analyses. A subgroup analysis was performed with employees who at 6 months’ follow-up reported to have been at risk of sick leave during the past 6 months (i.e. having experienced impaired work functioning and/or having considered sick leave) and/or have indeed been sick-listed over during the last 6 months. In the results section this subgroup will be referred to as the target group. Moreover, a per-protocol analysis was performed with employees who at 6 months’ follow-up reported to have discussed their (risk of) sick leave or impaired work functioning with their supervisor. Lastly, effect modification was tested, using a *p* value <.10 of the interaction term to indicate relevant effect modification. In case of effect modification, stratified analyses were performed. The statistical significance level was set at α = .05. All multilevel analyses were performed using MLwiN (version 2.28) [28]; all other analyses were performed using SPSS 22.0 [29].

## Results

### Flow of Study Participants

**Fig. 1:**
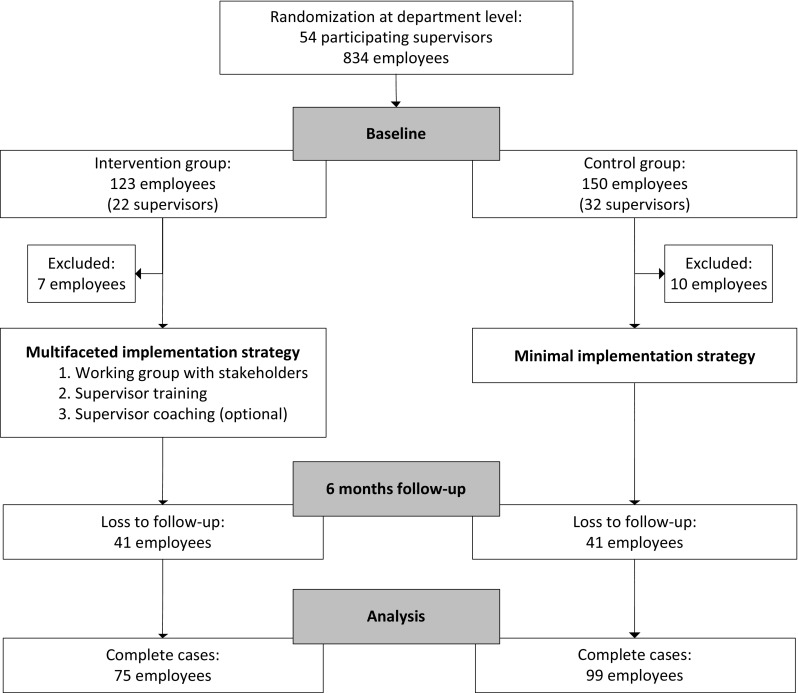
Participant flow

In Fig. 1, the flow of participants is presented. The 54 participating supervisors working in the university or university medical center were asked to approach their employees (N = 834) for participation. Assuming that all 834 employees were approached for participation by their supervisors, 33 % (N = 273) of approached employees participated at baseline. Loss to follow-up was 33 % in the intervention group and 27 % in the control group. In total, 75 employees in the intervention group and 99 employees in the control group were included in the analyses. The drop-out analysis showed that there were no significant differences in the primary outcome between completers and non-completers (social norms from organization *p* = .06; social norms from supervisor *p* = .73).

### Baseline Characteristics

Baseline characteristics of participants are presented in Table 1. Only one department of the university participated in the trial, and this department was randomly assigned to the intervention group. Thus, all participants in the control group where employed by the university medical center, while in the intervention group one-fifth of participants were employed by the university. The majority of participants were female, had a high level of education and a permanent employment contract.

**Table 1:** Baseline characteristics of the study population (n = 256)

	Intervention group (n = 116)	Control group (n = 140)
Organization		
University medical center, n (%)	91 (78 %)	140 (100 %)
University, n (%)	25 (22 %)	0 (0 %)
Female sex, n (%)	100 (86 %)	118 (84 %)
Age in years, M (SD)	42 (11)	44 (11)
High level of education (higher professional education or university), n (%)	61 (53 %)	94 (67 %)
Type of contract		
Permanent, n (%)	101 (89 %)	115 (85 %)
Temporary, n (%)	12 (11 %)	20 (15 %)
Working hours per week according to contract, M (SD)	30 (6)	27 (8)
Self-reported health condition		
Poor or moderate, n (%)	12 (11 %)	6 (5 %)
(Very) good or excellent, n (%)	98 (89 %)	124 (95 %)
Self-reported reduced work functioning due to health complaints in last 6 months, n (%)	31 (32 %)	32 (26 %)
Considered taking sick leave due to health complaints in last 6 months, n (%)	25 (26 %)	35 (28 %)
Took sick leave due to health complaints in last 6 months, n (%)	53 (55 %)	60 (48 %)

### Perceived Social Norms

As shown in Table 2, perceptions of social norms regarding supervisor collaboration at the organizational and at the supervisor level at baseline were relatively high in both groups. No significant difference over time was observed between the groups in the crude and adjusted multilevel analyses. In addition, the sub-group analysis with the target group and the per-protocol analysis also showed no significant difference over time between the intervention group and the control group.

**Table 2:** Mean scores on perceived social norms at baseline and 6 months’ follow-up and multilevel analysis results

All participants	Intervention group (n = 75)	Control group (n = 99)	ML model crude			ML model adjusted^a^		
	M (SD)	M (SD)	B (SE)		[95 % CI]	B (SE)		[95 % CI]
Perceived social norms from organization regarding joint problem-solving (range 1–5)								
Baseline	3.4 (.9)	3.3 (.9)						
6 months’ follow-up	3.3 (.9)	3.4 (.8)	−.06 (.13)		[−.32 to .20]	<−.01 (.14)		[−.28 to .28]
Perceived social norms from supervisors regarding joint problem-solving (range 1–5)								
Baseline	4.0 (.6)	3.8 (.7)						
6 months’ follow-up	3.8 (.6)	3.8 (.6)	−.12 (.11)		[−.33 to .09]	−.16 (.11)		[−.39 to .06]

^a^Confounders: age, sex, level of education, job insecurity, organization, general health, at risk for sick leave, sick leave, distress, need for recovery, decision authority, experienced leadership style (transformational leadership)

#### Effect Modification

Regarding social norms at the organization level, educational attainment and the extent to which participants experienced decision authority showed significant interaction terms. However, in subsequent stratified analyses, none of the subgroups showed significant differences between the intervention and the control group for social norms at the organizational level.

Regarding the perceptions of social norms at the supervisor level, sex, level of education and job insecurity showed significant interaction terms. Also for social norms at the supervisor level, there were no significant differences in the subgroup analysis between the intervention and control group.

### Other Outcomes

As presented in Table 3, for none of the other outcomes significant differences were observed over time between the intervention group and the control group. On all outcomes with a fixed score range, the average scores were relatively high at baseline and remained high at 6 month follow-up. In the intervention group, at baseline participants reported sick leave for 4.6 days in total during the previous 6 months, which decreased to 2.4 days at 6 month follow-up. In the control group, it was 3.8 days at baseline and remained approximately the same at 6 month follow-up. This difference between the groups was not statistically significant.

**Table 3:** Mean scores on other outcomes at baseline and 6 months’ follow-up and multilevel analysis results

	Intervention group (n = 75)	Control group (n = 99)	ML model crude		ML model adjusted^a^	
	M (SD)	M (SD)	B (SE)	[95 % CI]	B (SE)	[95 % CI]
Attitude regarding joint problem-solving (range 3–15)						
Baseline	12.7 (1.6)	12.2 (1.6)				
6 months’ follow-up	12.3 (1.5)	12.2 (1.5)	−.08 (.23)	[−.50 to .34]	−.24 (.23)	[−.69 to .21]
Self-efficacy regarding joint problem-solving (range 3–15)						
Baseline	12.3 (1.6)	11.8 (1.9)				
6 months’ follow-up	12.3 (1.7)	11.7 (1.9)	.41 (.26)	[−.10 to .92]	.45 (.29)	[−.12 to 1.02]
Intention to apply joint problem-solving (range 1–5)						
Baseline	4.0 (.8)	3.9 (.8)				
6 months’ follow-up	3.7 (.8)	3.8 (.7)	−.15 (.11)	[−.36 to .06]	−.21 (.12)	[−.45 to .02]
Self-efficacy regarding return-to-work (range 4–40)						
Baseline	31.3 (5.6)	30.8 (4.8)				
6 months’ follow-up	30.4 (5.6)	30.9 (4.8)	−.76 (.76)	[−2.24 to .72]	−.87 (.83)	[−2.49 to .75]
Number of episodes on sick leave in last 6 months						
Baseline	.9 (1.1)	.8 (1.2)				
6 months’ follow-up	.7 (1.2)	.6 (1.3)	.08 (.18)	[−.27 to .44]	−.05 (.20)	[−.44 to .34]
Total number of work days on sick leave during last 6 months						
Baseline	4.6 (12.9)	3.8 (9.4)				
6 months’ follow-up	2.4 (6.7)	3.6 (19.7)	−1.61 (2.35)	[−6.21 to 3.00]	−2.54 (2.73)	[−7.88 to 2.81]
Perceived supervisor support (range 4–16)						
Baseline	12.3 (1.9)	11.6 (2.0)				
6 months’ follow-up	11.3 (2.5)	11.5 (2.0)	−.52 (.40)	[−1.30 to .27]	−.22 (.32)	[−.86 to .41]
Satisfaction (range 1–5)						
Baseline	4.0 (1.1)	4.0 (1.0)				
6 months’ follow-up	3.8 (.9)	3.8 (1.0)	−.08 (.31)	[−.69 to .52]	−.25 (.35)	[−.94 to .43]

	n (%)	n (%)	B (SE)	[95 % CI]	B (SE)	[95 % CI]
Yes, discussed work functioning problems or risk of sick leave with supervisor						
Baseline	32 (60 %)	33 (55 %)				
6 months’ follow-up	23 (55 %)	23 (48 %)	−.10 (.13)	[−.36 to .16]	−.18 (.13)	[−.44 to .08]
Yes, discussed sick leave with supervisor						
Baseline	41 (77 %)	43 (72 %)				
6 months’ follow-up	23 (92 %)	27 (82 %)	.02 (.12)	[−.21 to .25]	.07 (.12)	[−.17 to .30]

^a^Confounders: age, sex, level of education, job insecurity, organization, general health, at risk for sick leave, sick leave, distress, need for recovery, decision authority, experienced leadership style (transformational leadership)

At baseline, 60 % of employees in the intervention group who reported work functioning problems or risk of sick leave and 55 % in the control group reported that they had discussed this with their supervisor. At 6 months’ follow-up, these percentages were 55 and 48 %, respectively. Regarding employees who had been on actual sick leave, 77 % in the intervention group and 72 % in the control group reported at baseline that they had discussed this with their supervisor. At 6 months’ follow-up, these percentages were 92 and 82 %, respectively. There were no statistically significant differences between the groups.

At baseline, eight participants reported that a third party had been present at a meeting between the participant and their supervisor (occupational health professional N = 1; HR professional N = 5; head of department N = 1; other supervisor N = 1). At 6 months’ follow-up, none of the participants reported that a third party had been present.

## Discussion

We investigated the effect of a multifaceted strategy to implement the participatory approach (PA) at supervisor level, aiming to further improve collaboration between supervisors and employees regarding work functioning problems due to health concerns. This study focuses on the effect on employees’ perceived social norms regarding the use of the PA to deal with work functioning problems, and on several other outcomes at employee level. Our findings indicate that the implementation strategy had no effect on employee perceptions of social norms, attitude, self-efficacy and intention regarding joint problem-solving with supervisors to improve work functioning and perceived supervisor support.

Supervisors were positioned as key players in this organizational intervention, and it was hypothesized that supervisors would be encouraged to promote discussions on work functioning problems due to health complaints with their employees. We therefore reasoned that an effect could be expected on employees’ perceptions of social norms at the organizational or supervisor level regarding joint problem-solving of work functioning problems. However, such an effect was not found, even in the subgroup of participants who had discussed their (risk of) sick leave with their supervisors during the follow up time of the study. Apparently, a strategy to implement an organizational intervention of this type at the supervisory level does not lead to measurable changes in the perceptions of employees overall.

Our results show that at baseline, only 60 % of participating employees with work functioning problems or risk of sick leave in the intervention group reported that they had discussed this with their supervisor. In the control group this was 55 %. At 6 month follow-up, the percentages were 55 and 48 %, respectively. For participating employees who had been on sick leave, 77 and 72 %, respectively, reported at baseline that they had discussed this with their supervisor and 92 and 82 % at 6 month follow-up, respectively. The goal of the PA intervention strategy was to encourage supervisors to be more proactive in cases of work functioning problems or at risk of sick leave, as well as, in cases of actual sick leave. It seems, however, that discussing and dealing with work functioning problems or sick leave remained difficult for employees and their supervisors. A possible explanation for this difficulty is the current economic climate, in which fear for losing one’s job is conceivably greater than in a better economic climate. Nevertheless, the implementation strategy was evidently unsuccessful in increasing employees’ inclination to discuss work functioning problems with their supervisor. Employees and supervisors had the possibility to ask an occupational health professional to act as process leader in the meetings between both parties. At 6 months’ follow-up, none of the participants reported that a third party had been present at a meeting between the participant and their supervisor. Apparently, supervisors and employees did not seek assistance for the meetings.

Furthermore, satisfaction regarding discussing work functioning problems or being at (risk of) sick leave did not increase over time, and perceived supervisor support in the intervention group was even slightly lower after 6 months compared to baseline. Perhaps the training in application of the PA resulted in more businesslike conversations between supervisors and employees, at the expense of showing empathy.

Although applying the PA for employees at risk of sick leave was an innovative approach of our study, applying the PA for employees on sick leave has been studied before. Previous studies have shown that the PA is effective in improving RTW and shortening the duration of sick leave [5–9]. In our study, the duration of sick leave decreased from an average of 4.6–2.4 days between baseline and 6 months’ follow-up in the intervention group while it remained similar in the control group (3.8–3.6 days). The difference between the groups was not statistically significant, perhaps due to low power.

Discussing work functioning problems due to health complaints may lead to deciding that some kind of work adjustment is desirable to maintain good work functioning. Exploratory analyses showed that when work functioning problems were discussed with the supervisor, in around half of the cases this led to some kind of work adjustment. Most often reported work adjustments were adjustments in working hours, adjustments of tasks and responsibilities, and adjustments in the amount of work. As implementing work adjustments for employees with chronic health conditions is associated with a decrease in sick leave [30], this is a positive finding.

### Study Limitations

Several limitations should be addressed. First, supervisors participating in the study were asked to invite their employees to fill out the questionnaires. Although this strategy might increase participation rates, it meant that the recruitment of employees depended on the willingness of supervisors to recruit. Furthermore, this strategy may have led to selection bias, as some supervisors may have been more inclined than others to ask their employees to participate. Any effect of this bias and its direction are difficult to estimate.

Second, this study of effects of the PA implementation strategy at employee level suffered a high loss to follow-up of around 30 % of the initial participants. We do not know the reasons for loss to follow-up. Some conceivable reasons might be that the participant did not work in the organization anymore at 6 months’ follow-up or that they were dissatisfied with the way PA was applied with them (if it was applied at all). Loss to follow-up occurred in both the intervention and the control group. A drop-out analysis between completers and non-completers showed no statistically significant differences regarding perceived social norms.

Third, the RCT was performed in three organizations: a university, a university medical center and a steel factory. Due to difficulties in organizing this part of the trial, it was not possible to recruit employees of the steel factory for the present study of effects regarding outcomes at employee level. In addition, only one department of the university participated in the trial. Because randomization was carried out at the department level, this meant that the university could only be represented in one of the study groups. The university was randomly assigned to the intervention group. This may have led to the intervention group and control group being less comparable, as it is conceivable that a university manages (risk of) sick leave differently than a medical center.

Lastly, some limitations should be mentioned regarding our methods of measurement. Perceived social norms from the organization and the supervisor were both measured using one self-formulated non-validated item. In future work, it might be valuable to develop and validate a more robust measure of perceived social norms. In addition, because the Attitude-Social Influence-Self-efficacy (ASE) model is meant to explain (intented) behavior, it not developed as a theoretical framework to explore differences on behavioral outcomes/predictors at employee level [21]. Furthermore, sick leave data were based on self-reports, which may have led to bias. Although previous research has found that self-reported data on sick leave closely corresponds to administrative data [31], this does not take into account that taking part in a study aiming to prevent or reduce sick leave might cause participants to unconsciously underestimate their sick leave at 6 months’ follow-up. In addition, it is conceivable that taking part in this study caused participants to become more aware of any work functioning problems. Nevertheless, if this was the case, it did not seem to have had an effect on the percentage of participants who discussed work functioning problems or risk of sick leave with their supervisor as this percentage was even (non-significantly) lower after 6 months than at baseline.

## Conclusion

The multifaceted strategy to implement the PA did not show effects on outcomes at employee level such as perceived social norms from their organization and their supervisor, self-efficacy and intention regarding joint problem-solving to improve work functioning and perceived supervisor support. To gain effects at employee level the present implementation strategy cannot be recommended. The implementation strategy should be extended to target not only the organization and supervisors but also the employees themselves, for instance by giving them a short training with regard to the PA. In addition, the organization should increase employees’ awareness of, and more clearly propagate the participatory approach as their method of tackling work functioning problems due to health problems.
